# Clinical characteristics and management of immune checkpoint inhibitor-related cardiotoxicity: A single-center experience

**DOI:** 10.3389/fcvm.2023.1093383

**Published:** 2023-04-06

**Authors:** Junjuan Xiao, Xingyu Li, Xuan Wang, Yaping Guan, Hairong Liu, Jing Liang, Yan Li, Baocheng Wang, Jun Wang

**Affiliations:** ^1^ Department of Oncology, The First Affiliated Hospital of Shandong First Medical University & Shandong Provincial Qianfoshan Hospital, Jinan, China; ^2^ Shandong Key Laboratory of Rheumatic Disease and Translational Medicine, Jinan, China; ^3^ Shandong Lung Cancer Institute, Jinan, China; ^4^ 960 Hospital of the People’s Liberation Army, Jinan, China

**Keywords:** cardiotoxicity, immune checkpoint inhibitor, PD-1, PD-L1, immune-related adverse event, myocarditis

## Abstract

**Background:**

Immune checkpoint inhibitors (ICIs) have revolutionized cancer therapy in the past decade and amplify T-cell-mediated immune responses by disrupting immunoinhibitory signals. The augmented T-cell immune response has led to a range of immune-related adverse effects (irAEs). Immune-related cardiotoxicity has been reported in case series but has been underappreciated due to difficulties in diagnosis. This article describes epidemiological, clinical presentation, subtype, and treatment data and a new systematic framework for the clinical management of cardiotoxicity.

**Methods:**

Data were extracted for cancer patients who received ICIs in a single center between January 1, 2020, and February 28, 2022. ICI-associated cardiotoxicity was clinically diagnosed based on clinical presentations, biochemical biomarkers, and imaging features.

**Results:**

We identified a total of 12 (2.46%) cases of ICI-related cardiotoxicity from 487 patients who received PD-1 or PD-L1 inhibitors. All patients were diagnosed with advanced or metastatic solid tumors. The severity of ICI-related cardiotoxicity ranged from subclinical cardiac abnormalities (subclinical type) with only asymptomatic troponin-I (TnI) elevations (25.0%) to symptomatic cardiac abnormalities (clinical type) (75.0%). Patients with symptomatic cardiac abnormalities had several manifestations, including tachyarrhythmia (16.7%), bradyarrhythmia (41.7%), or cardiac failure (8.3%). The median immunotherapy exposure time was 1.5 doses (range: 1 to 5), and the median time from the initial immunotherapy to the onset of ICI-related cardiotoxicity was 33.5 days (IQR: 20.3 to 46.8). Most patients, including those with subclinical cardiac abnormalities, were administered systemic corticosteroids (58.3%). One (8.3%) patient was put on mechanical ventilation, one (8.3%) received plasma exchange therapy, one (8.3%) was implanted with a pacemaker, and one (8.3%) was admitted to the ICU. Three patients with symptomatic cardiac abnormalities (25.0%) died, and other patients presented with significant clinical improvement with good outcomes.

**Conclusion:**

ICI-related cardiotoxicity is uncommon but critical with a high mortality rate and poor prognosis, especially for a small group of patients with symptomatic cardiac abnormalities. More attention should be given to cardiotoxicity associated with ICIs, and these patients should be given baseline examinations and biochemical analyses before and after the initiation of immunotherapy, intensive cardiac assessments, an accurate and rapid diagnosis, and timely multidisciplinary management with immunosuppressive agents and other necessary clinical interventions.

## 1. Introduction

Antineoplastic drugs have evolved and developed remarkably, from chemicals to molecular targeted agents and immune checkpoint inhibitors (ICIs), in the past decade. ICIs, known as the leading type of cancer immunotherapy, are monoclonal antibodies that reactivate T lymphocytes by blocking immunoinhibitory signals. Representative ICIs targeting two pathways regulated by programmed cell death protein-1 (PD-1) and its ligand programmed cell death ligand-1 (PD-L1) and cytotoxic T-lymphocyte-associated protein 4 (CTLA-4) have been successfully developed (1, 2). Several ICIs, including ipilimumab, nivolumab, pembrolizumab, atezolizumab, durvalumab, and avelumab, have received approval from the U.S. Food and Drug Administration (FDA), European Medicines Agency (EMA), or National Medical Products Administration (NMPA) for use in several advanced cancers, including non-small cell lung cancer (NSCLC), melanoma, urothelial cancer, refractory Hodgkin's lymphoma, liver cancer, and any solid tumor with a microsatellite instability-high (MSI-H), mismatch repair deficient (dMMR) profile, or tumor mutational burden-high (TMB-H).

Despite the rapid advances in the field of cancer immunotherapy, system-wide toxicities associated with ICIs have been noted and have been termed immune-related adverse events (irAEs). The most common irAEs are dermatologic, endocrine, hepatic, and gastrointestinal toxicities. Basically, these irAEs are reversible and can be controlled with basic corticosteroid therapy (3). Cardiotoxicities, such as myocarditis, electrical dysfunction, acute coronary syndrome, pericarditis, and acute systolic heart failure, are among the rare organ toxicities associated with ICIs,. With the increased use of immunotherapy alone or in combination with other agents across various human malignancies, cardiac adverse events are usually reported and have attracted much attention. Myocarditis is the most common cardiotoxicities occurring following ICI treatment. However, myocarditis sometimes develops into a potentially fatal clinical disease, especially when ventricular arrhythmias or heart failure occurs (4). Due to the low frequency and subtle features of the initial clinical presentations of cardiac irAEs, these cases are usually missed on subsequent initial presentation, despite being obvious, or are underappreciated due to the nonspecific clinical manifestations (5). In addition, clinical risk factors associated with ICI-related cardiotoxicities have not been identified, although patients with the combination of CTLA-4 and PD-1 inhibition are prone to develop serious cardiotoxicities (6). Here, we presented a series of ICI-related cardiotoxicity cases at a single center. These patients with diverse clinical presentations had uncommon cardiac irAEs after ICI therapy and were diagnosed and managed precisely according to their classification and grade.

## 2. Materials and methods

### 2.1. Patients

All cases of ICI-related cardiotoxicity were derived from the Department of Oncology, The First Affiliated Hospital of Shandong First Medical University & Shandong Provincial Qianfoshan Hospital, between January 1, 2020, and February 28, 2022. Clinical data, including standard demographics, cardiovascular risk factors, clinical symptoms, signs, medications, laboratory findings, electrocardiograms (ECGs), echocardiographic variables, and outcomes, were retrieved from the hospital's electronic medical records. Written informed consent for publication was provided by all patients and was in accordance with the Helsinki Declaration. Approval was obtained from the independent research ethics of The First Affiliated Hospital of Shandong First Medical University & Shandong Provincial Qianfoshan Hospital (NO: YXLL-KY-2020-007) to publish the case details and conduct this research. Two physicians independently reviewed the clinical data.

### 2.2. Diagnosis of ICI-related cardiac irAEs

ICI-related cardiotoxicity was diagnosed by a local multidisciplinary team including cardiovascular specialists, imaging specialists, pathologists and oncologists based on several clinical features according to the previously proposed criteria by Marc *P*. Bonaca et al. (7). The clinical features included clinical signs, symptoms, biochemical biomarkers (TnI, MYO, CK, CK-MB, BNP, and LDH), and imaging such as electrocardiogram, echocardiography, cardiac magnetic resonance (CMR), and cardiac ^18^F-fluorodeoxyglucose (^18^F-FDG) PET/CT. However, none of the patients included in the case series underwent invasive endomyocardial biopsy or autopsy.

The diagnosis depended mainly on clinical features, including the signs and symptoms, biomarkers, electrocardiogram and echocardiography, and prior medication history of ICIs. Subclinical cardiac abnormalities refer to asymptomatic TnI elevations alone, whereas patients with symptomatic cardiac abnormalities were those who had several manifestations, including tachyarrhythmia, bradyarrhythmia, or cardiac failure, with TnI elevations and other positive laboratory findings and signs.

## 3. Results

### 3.1. Clinical characteristics of patients with cardiac irAEs

A total of 487 patients received immunotherapy with PD-1 or PD-L1 inhibitors in the Department of Oncology, the First Affiliated Hospital of Shandong First Medical University. The common indications for ICI treatment included NSCLC, small cell lung cancer, hepatocellular carcinoma, esophageal carcinoma, gastric carcinoma, and melanoma. None of the patients had a prior history of autoimmune or allergic disease or any acute heart diseases or corresponding symptoms. All of the patients' baseline left ventricular ejection fraction (LVEF), laboratory findings, and ECG were normal. The total incidence of irAEs was 51.7% (252/487), and 44 patients (9.0%) developed severe irAEs, including 22 patients (4.5%) with pneumonia, 13 patients (2.7%) with hematotoxicity, and 8 patients (1.6%) with cardiotoxicity. In addition, one patient (0.2%) developed severe erythrodermic psoriasis.

We identified 12 (2.46%) patients with ICI-related cardiotoxicity (Table 1). As shown in Table 2, 8 patients (66.7%) were male. The median age was 67.5 years old (range: 58.3 to 71.8). Three patients were diagnosed with NSCLC, and 2 were diagnosed with gastric cancer. Other malignancies included gallbladder cancer, breast cancer, colon cancer, cholangiocarcinoma, pancreatic cancer, thymic cancer, and cervical cancer. Before the initiation of immunotherapy, 41.7% of the patients underwent surgery, and 83.3% of the patients received chemotherapy. A total of 16.7% of the patients received previous radiotherapy, and antiangiogenic agents were administered in 33.3% of the patients. Only 3 patients had a history of hypertension, and 6 patients were diagnosed with diabetes. Although 2 patients had a prior diagnosis of chronic coronary heart disease without corresponding symptoms, they did not receive pre-ICI home cardiovascular medications, including statins, aspirin, beta-blockers, angiotensin-converting enzyme inhibitors, angiotensin receptor blockers, or calcium-channel blockers.

**Table 1:** Summary of patients with ICI-related cardiac irAE at our center.

Cases	Tumor type	Treatment of ICIs	PD-L1 expression	Cycles of ICI treatment	Concurrent other irAE	Cardiac irAE	Grade	Clinical presentation	Alterative laboratory tests	Alterative echocardiographic datas	Alterative radiologic findings	Treatment of cardiac irAE	Prognosis
Patient 1	NSCLC	Tislelizumab	1%	5	None	Myocarditis	1	Asymptomatic	TnI and TnT elevation	NA	NA	Observation	Alive
Patient 2	Pancreatic carcinoma	Sintilimab	0%	5	None	Myocarditis and Pericarditis	1	Asymptomatic	TnI elevation	Small pericardial effusion	NA	Observation	Alive
Patient 3	Breast cancer	Clinical trial	Unknown	1	Hepatic	Myocarditis and Pericarditis	2	Asymptomatic	TnI, TnT and LDH elevation	Pericardial effusion	left pleural effusion and pericardial effusion	2 mg/kg methylprednisolone and IVIG	Alive
Patient 4	Gastric carcinoma	Sintilimab	Unknown	1	Dermatologic	Myocarditis and Pericarditis	1	Fatigue and dermatologic	TnI elevation	Pericardial effusion;thickened left ventricular wall	NA	1 mg/kg methylprednisolone	Dead
Patient 5	Thymic carcinoma	Camrelizumab	0%	2	Dermatologic And Skeletal muscle	Myocarditis	3	Blepharoptosis and chest tightness	MYO, TnI, CK and LDH elevation	Left atrium distension; thickened left ventricular wall;aortic valve degeneration	NA	1,000 mg methylprednisolone and IVIG	Alive
Patient 6	Bladder cancer	Tislelizumab	Unknown	1	None	Myocarditis	5	Exertional dyspnea	BNP, MYO, TnI, CK and LDH elevation	NA	NA	4 mg/kg methylprednisolone	Dead
Patient 7	Gastric carcinoma	Tislelizumab	unknown	2	Skeletal muscle	Myocarditis	4	Palpitation, chest tightness and myalgia	CK, LDH, MYO, BNP and TnI elevation	NA	NA	500 mg methylprednisolone	Alive
Patient 8	Cholangiocarcinoma	Sintilimab	Unknown	1	Skeletal muscle	Myocardiits	3	Palpitation, chest tightness and fatigue, Blepharoptosis	LDH, MYO, BNP and TnI elevation	NA	Left pleural effusion	4 mg/kg methylprednisolone and tacrolimus	Alive
Patient 9	NSCLC	Pembrolizumab	0%	2	Skeletal muscle	Myocarditis and Pericarditis	4	Exertional dyspnea	TnI, TnT, BNP and LDH elevation	Pericardial effusion	Bilateral pleural effusion and pericardial effusion	500 mg methylprednisolone and IVIG	Dead
Patient 10	Colon cancer	Toripalimab	Unknown	1	None	Myocarditis	5	Palpitation and chest tightness	TnI, MYO, BNP and CK-MB elevation	NA	NA	4 mg/kg methylprednisolone	Dead
Patient 11	NSCLC	Sintilimab	Unknown	1	None	Myocarditis and Pericarditis	5	Palpitation and chest tightness	TnI, MYO, CK and CK-MB elevation	Pericardial effusion	NA	None	Dead
Patient 12	Cervical squamous cell carcinoma	Sintilimab	Unknown	2	Skeletal muscle	Myocarditis	4	Palpitation, chest tightness and fatigue, Blepharoptosis	TnI, MYO, LDH, CK and CK-MB elevation	Mitral regurgitation	NA	1,000 mg methylprednisolone, IVIG and tacrolimus	Alive

ICI, immune checkpoint inhibitor; irAE, immune-related adverse event; NSCLC, Non-small cell lung cancer; TnT, Troponin T; TnI, Troponin I; BNP, B natriuretic peptide; CK, creatine kinase; LDH, lactate dehydrogenase; LVEF, left ventricular ejection fraction; IVIG, intravenous immunogloblin.

**Table 2:** Basic line clinical characteristics of patients with ICI-related cardiac irAE.

Characteristics	Patients (*n*), Median (IQR), No. (%)
**Age, median (IQR), year**	67.5 (58.3–71.8)
**Sex**	
Male	8 (66.7%)
Female	4 (33.3%)
**Tumor type**	
Non-small cell lung cancer	3 (25.0%)
Gastric cancer	2 (16.7%)
Gallbladder cancer	1 (8.3%)
Breast cancer	1 (8.3%)
Colon cancer	1 (8.3%)
Cholangiocarcinoma	1 (8.3%)
Pancreatic cancer	1 (8.3%)
Thymic cancer	1 (8.3%)
Cervical cancer	1 (8.3%)
**Prior Treatment**	
Operation	5 (41.7%)
Chemotherapy	10 (83.3%)
Radiotherapy	2 (16.7%)
Anti-angiogenic agents	4 (33.3%)
Immunotherapy	0 (0.0%)
**Co-morbidities**	
Hypertension	3 (25.0%)
Diabetes	6 (50.0%)
Coronary heart disease	2 (16.7%)
**Types of ICI**	
Nivolumab	0 (0.0%)
Pembrolizumab	1 (8.3%)
Sintilimab	5 (41.7%)
Tislelizumab	3 (25.0%)
Toripalimab	1 (8.3%)
Camrelizumab	1 (8.3%)
Other	1 (8.3%)
**ICI combination**	
Yes	11 (91.7%)
No	1 (8.3%)
**Administration of ICI in our hospital**	
Yes	10 (83.3%)
No	2 (16.7%)
**Types of cardiac irAE**	
Myocarditis	12 (100.0%)
Others	0 (0.0%)
**Grade of cardiac irAE**	
G1 (asymptomatic)	3 (25.0%)
G2	1 (8.3%)
G3–4	5 (41.7%)
G5	3 (25.0%)
Median immunotherapy exposure (cycles)	1.5 (1–5)
Days from the initial immunotherapy to onset of cardiac irAE	33.5 (20.3–46.8)
**Initial clinical presentations**	
No symptoms and signs	3 (25.0%)
Fever	1 (8.3%)
Chest tightness	6 (50.0%)
Palpitation	5 (41.7%)
Dyspnea	2 (16.7%)
Myalgia	1 (8.3%)
Fatigue	3 (25.0%)
Myasthenia	4 (33.3%)
Blepharoptosis	3 (25.0%)
**Concurrent other irAE**	
Dermatologic	2 (16.7%)
Endocrine	0 (0.0%)
Hepatic	1 (8.3%)
Gastrointestinal	0 (0.0%)
Pulmonary	0 (0.0%)
Skeletal muscle	5 (41.7%)

IQR, interquartile range; ICI: immune checkpoint inhibitor; irAE, immune-related adverse event.

As shown in Table 2, the most frequently used ICIs were sintilimab (41.7%) and tislelizumab (25.0%). No patients were treated with PD-L1 inhibitors. The median immunotherapy exposure was 1.5 doses (range: 1 to 5). The median time from the initial immunotherapy to the onset of ICI-related cardiotoxicity was 33.5 days (IQR: 20.3 to 46.8), with 66.7% of the ICI-related cardiotoxicities presenting within 42 days of ICI initiation. The clinical symptoms and signs at the time of diagnosis varied. The most common patient complaint was chest tightness (50.0%). Patients also frequently presented with complaints of palpitation (41.7%), fatigue (25.0%), and dyspnea (16.7%). Neuromuscular symptoms such as myasthenia/myositis (33.3%), blepharoptosis (25.0%), and myalgia (8.3%) also occurred in the patients with cardiac irAEs. In addition, some patients presented with other simultaneous irAEs, including dermatologic (16.7%) and hepatic (8.3%) toxicities. Four patients (33.3%) developed myocarditis with concurrent myositis. The percentages of the patients with G1, G2, G3-4, and G5 cardiac irAEs were 25.0%, 8.3%, 41.7%, and 25.0%, respectively. Overall, 3 patients had subclinical cardiac abnormalities, and 9 had symptomatic cardiac abnormalities.

**Figure 1:**
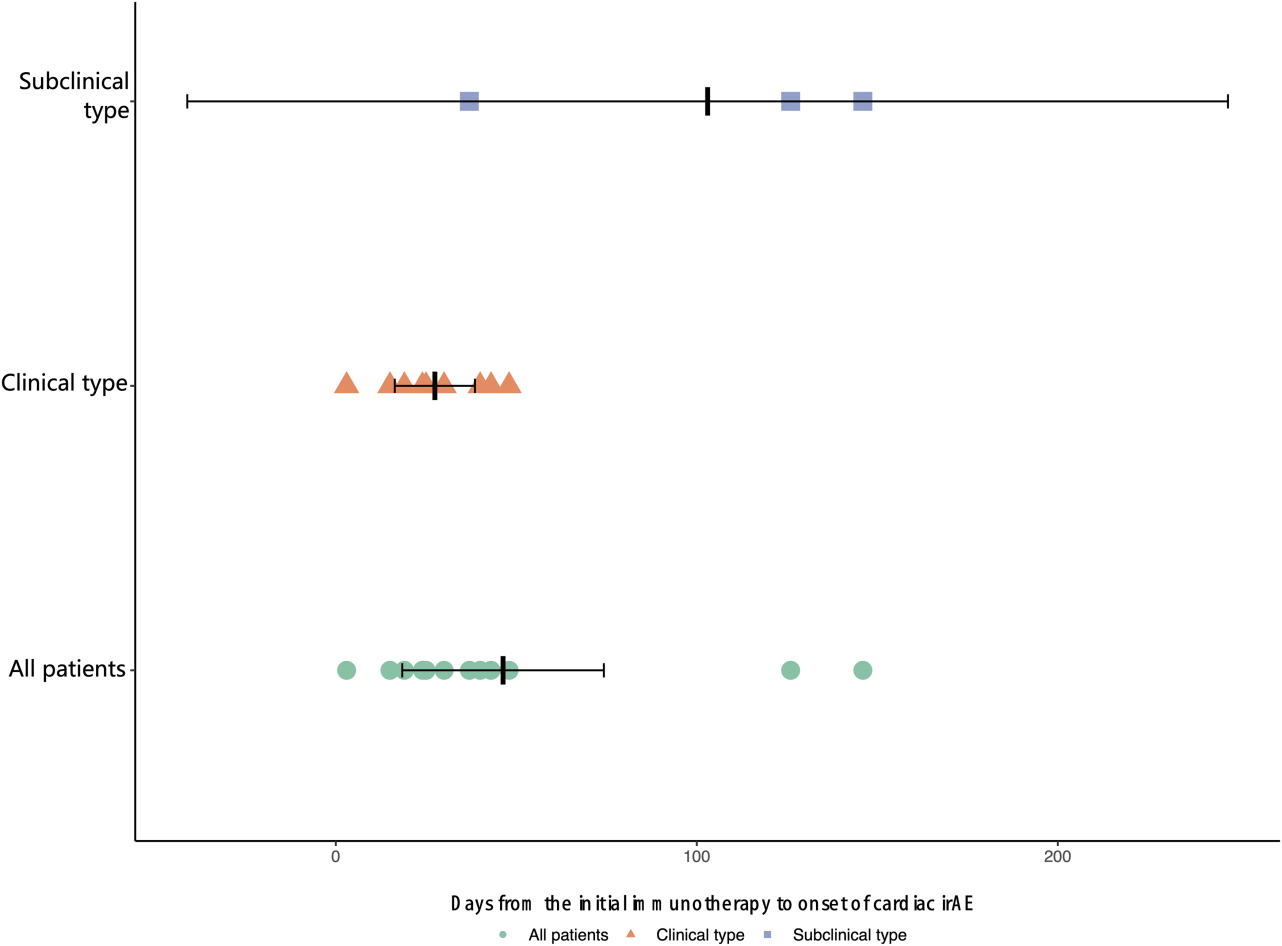
Time from the initial immunotherapy to onset of cardiac irAEs.

The median time from the initial immunotherapy to the onset of ICI-related cardiotoxicity for the subclinical type and clinical type was 126.0 days and 25.0 days (IQR: 17.0 to 41.5 days), respectively (Figure 1). Four patients with severe ICI-related cardiac irAEs had concurrent myositis, two had subclinical dermatologic irAEs, and one had severe hepatic irAEs. The patients with grade 2–5 ICI-related cardiac irAEs received steroid therapy.

### 3.2. Laboratory tests of patients with cardiac irAEs

We reviewed the patients' laboratory tests, including complete blood count, blood biochemical analysis, electrocardiogram, echocardiogram, and radiographic findings at the time of diagnosis of a cardiac irAE (Table 3). Lymphopenia was frequently found in the patients with cardiac irAEs (66.7%). We also calculated the neutrophil to lymphocyte ratio (NLR) and platelet to lymphocyte ratio (PLR) and found that an elevated NLR (>5) was observed in 7 patients (58.3%), and an elevated PLR (>185) was observed in 8 patients (66.7%). All patients had elevations in their TnI levels (100%), 7 patients had elevated LDH levels (58.3%), 5 had elevations in their CK (41.7%), 7 had elevations in their CK-MB levels (58.3%), 6 had elevated BNP levels (50.0%), and 7 had elevations in their MYO levels (58.3%). One or more elevated biomarkers were shown in the patients with subclinical cardiac abnormalities (Table 1). All of the patients with symptomatic cardiac abnormalities showed more elevated biomarkers simultaneously, and 5 patients also had concurrent elevated BNP levels. Nine patients (75.0%) had an aberrant ECG, but only 2 patients had an abnormal LVEF (16.7%). We identified 5 patients with pericardial effusion and 3 with pleural effusion through x-ray or chest computed tomography (CT).

**Table 3:** Laboratory tests and other tests for patients with cardiac irAE.

Laboratory tests	Baseline (median, range)	At the time of diagnosis of cardiac irAE (median, range)	Dynamics (%)
**Complete blood count**			
Neutrophils, ×10^9^/L	5.24 (3.59–6.31)	5.92 (3.08–9.19)	6 (50.0%)
Lymphocyte count, ×10^9^/L; Increased	1.15 (0.90–1.42)	0.92 (0.46–1.14)	8 (66.7%)
Eosinophils, ×10^9^/L; Increased	0.08 (0.02–0.11)	0.01 (0.00–0.05)	7 (58.3%)
Platelets, ×10^9^/L	208 (128–354)	229 (155–412)	
NLR; >5			7 (58.3%)
PLR; >185			8 (66.7%)
**Blood biochemical analysis**			
ALT, U/L	15.8 (10.1–31.1)	57.1 (136–325.8)	6 (50.0%)
AST, U/L	19.1 (11.9–25.2)	85.0 (23.7–229.1.0)	7 (58.3%)
LDH, U/L	216 (189.3–229);	455.0 (223.5–917.8)	7 (58.3%)
CK, U/L	57.0 (40.0–134.0)	69.0 (42.0–1559.0)	5 (41.7%)
CK-MB; ng/ml	17.4 (9.5–21.6)	53.4 (15.7–77.8)	7 (58.3%)
MYO, U/L	46.1 (24.9–74.3)	500.0 (42.9–853.6)	7 (58.3%)
TnI, ng/L	0.26 (0.12–0.40)	0.6 (0.12–8.13)	12 (100.0%)
BNP, pg/ml	32.5 (18.3–81.7)	133.4 (31.8–373.5)	6 (50.0%)
FERR, ng/ml	140.8 (52.1–585.0)	337.9 (94.4–1740.3)	8 (66.7%)
CRP, mg/L	3.12 (3.12–7.73)	15.8 (3.1–60.7)	8 (66.7%)
Positive autoantibodies			4 (33.3%)
**Electrocardiogram**			
Normal			3 (25.0%)
Sinus tachycardia			1 (8.3%)
Atrial flutter			1 (8.3%)
Ventricular Tachycardia			1 (8.3%)
Sinus bradycardia			1 (8.3%)
Atrioventricular block			1 (8.3%)
Bundle branch block			2 (16.7%)
Cardiac arrest			1 (8.3%)
ST segment elevation			3 (25.0%)
**Echocardiogram**			
LVDD, mm	43.8 (39.5–55.6)	40.7 (39.0–45.3)	
LVSD, mm	29.0 (25.4–34.4)	28.1 (24.2–32.8)	
E, cm/s	72.0 (66.0–80.0)	76.5 (53.8–83.8)	
A, cm/s	39.0 (30.0–50.0)	55.0 (42.3–77.5)	
E/A	1.9 (1.5–2.4)	1.5 (0.8–1.9)	
E’, cm/s	8.2 (6.7–10.8)	6.6 (4.4–9.8)	
E/E'	8.9 (7.7–11.6)	13.6 (8.9–16.2)	
LVEF; Reduced	67.5 (64.3–75.0)	64.0 (53.3–65.8)	2 (16.7%)
Cardiac dilatation			1 (8.3%)
Wall motion abnormality			1 (8.3%)
**Radiographic findings**			
Cardiac enlargement			1 (8.3%)
Pericardial effusion			5 (41.7%)
Pleural effusion			3 (25.0%)

NLR, neutrophil to lymphocyte ratio; PLR, platelet to lymphocyte ratio; ALT, alanine transaminase; AST, aspartate transaminase; LDH, lactate dehydrogenase; CK, Creatine kinase; CK-MB, Cardiac Creatine Kinase; MYO, myohemoglobin; TNI, troponin I; BNP, B-type natriuretic peptide; FER, Ferritin; CRP, C-reactive protein; ESR, erythrocyte sedimentation rate; LVDD, Left ventricular end diastolic diameter; LVSD, left ventricular systolic diameter; E, E peak of early diastolic mitral valve; A, A peak of ventricular diastole; E/A, Mitral valve diastolic flow spectrum; E/E’, Doppler spectrum of mitral annulus lateral wall tissue; LVEF, Left ventricular ejection fraction.

### 3.3. Clinical treatment and outcome

**Figure 2:**
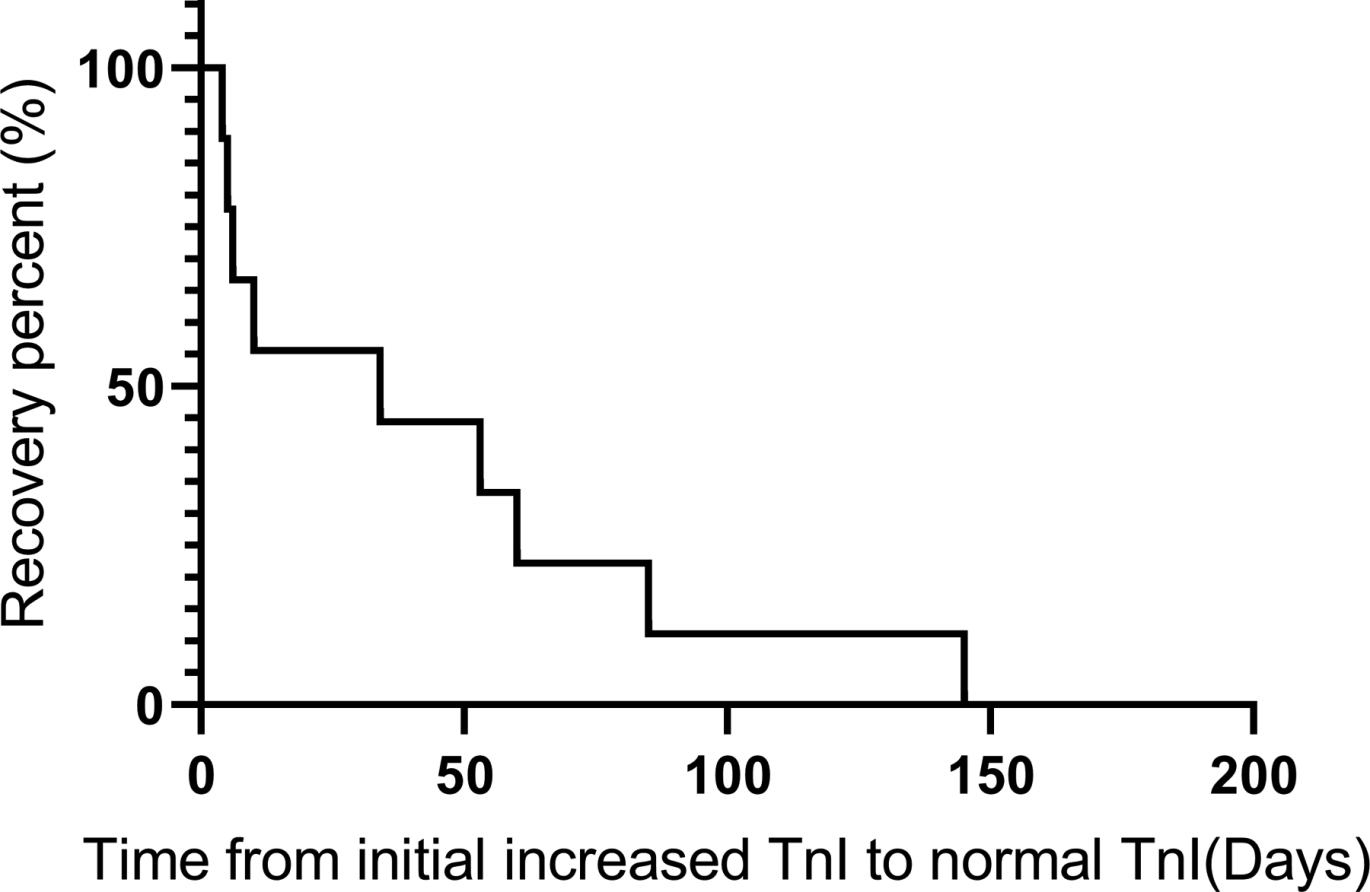
Recovery time from when the TnI level was initially increased to when a normal TnI level was obtained following corticosteroid and immune inhibitor administration.

We also summarized the clinical treatment and outcome of the patients with cardiac irAEs. As shown in Table 4, a total of 7 patients received systemic corticosteroid therapy (58.3%). The majority of the patients were treated with high-dose corticosteroids (58.3%). Four patients were administered 500 to 1,000 mg of methylprednisolone daily because of multiple life-threatening complications (Table 1). The median time of initial systemic corticosteroid use was 5.0 days (range: 0.3 to 17.3) after symptom onset or the detection of abnormal serum markers. The median duration of systemic corticosteroid administration was 9.0 weeks (range: 4.0 to 13.0). Moreover, 3 patients were prescribed intravenous immunoglobin (IVIG) (25.0%). Two patients received tacrolimus treatment (16.7%) due to the ineffectiveness of systemic corticosteroid treatment. None of the patients were administered other immune inhibitors, including anti-TNF-α antibody, anti-IL-6 antibody, the cytotoxic T-lymphocyte-associated antigen-4 (CTLA-4) fusion protein, and anti-CD52 antibody. The median time from the initial increase in TnI to normal TnI following corticosteroid and immune inhibitor administration was 34.0 days (IQR: 5.5 to 72.5) (Figure 2).

**Table 4:** Clinical treatment and outcome for patients with cardiac irAE.

Treatment	Median (IQR); No. (%)
**Medicine**	
Steroids	7 (58.3%)
Immunoglobin	3 (25.0%)
Anti-TNF-α	0 (0.0%)
Anti-IL-6	0 (0.0%)
MMF	0 (0.0%)
Tacrolimus	2 (16.7%)
**Advanced treatment**	
Mechanical ventilation	1 (8.3%)
ECMO	0 (0.0%)
Pacemaker	1 (8.3%)
Plasma exchange	1 (8.3%)
Admission to ICU	1 (8.3%)
**Evaluation of clinical presentation**	
Improvement	9 (75.0%)
Worse	3 (25.0%)
**Clinical outcome**	
Discharge from hospital	9 (75.0%)
Death	3 (25.0%)
Time of initial systemic corticosteroid use after symptom onset or detection of abnormal serum markers, days	5.0 (0.3–17.3); 8 (66.7%)
Duration of systemic corticosteroid administration, weeks	9.0 (4.0–13.0); 7 (58.3%)
Time from initial increased TnI to normal TnI, days	34.0 (5.5–72.5); 9 (75.0%)
Time from initial presentation to clinical improvement, days	22.0 (4.5–26.0); 6 (50.0%)
Time of hospital stay	14.0 (5.0–21.5); 9 (75.0%)
Time from initial diagnosis of cardiac irAE to death, days	2.0 (1.0–5.0); 3 (25.0%)
Rechallenge with ICIs	3 (25.0%)

MMF, Mycophenolate mofetil; ECMO, extracorporeal membrane oxygenation; ICU, intensive care unit.

The patients who progressed on prior treatment and had life-threatening complications were admitted to the ICU (8.3%) or treated with mechanical ventilation (8.3%), plasma exchange therapy (8.3%), or an implanted pacemaker (8.3%). No patients received extracorporeal membrane oxygenation (ECMO). Death occurred in 3 (25.0%) patients, with a median time from the initial diagnosis of cardiac irAE to death of 2.0 days (range: 1.0 to 5.0). Another 9 patients (75.0%) were finally discharged from the hospital, with a median time from the initial diagnosis of cardiac irAEs to clinical improvement of 29.0 days (IQR: 8.0–40.3) and a median hospital stay of 14.0 days (range: 5.0 to 21.5). ICIs were rechallenged in 3 patients with subclinical cardiac irAEs (25.0%).

The cumulative incidence of disease progression was 58.3%, 75.0%, and 83.3% at 6 months, 12 months, and 24 months, respectively. Disease control as defined by SD, PR, and CR was achieved in 8 patients (75.0%), including 7 patients with SD and 1 patient with PR (Table 5). Overall, 8 patients still survive now. However, one patient with the subclinical type died of a tumor, and three patients with severe irAEs died of cardiotoxicity. The median PFS and OS for the patients with cardiac irAEs were 4.0 and 18.0 months, respectively.

**Table 5:** The efficacy with ICIs for patients with cardiac irAE.

Efficacy assessment	Number (%)
Complete response	0 (0.0%)
Partial response	1 (11.1%)
Stable disease	7 (77.8%)
Disease progression	1 (11.1%)

## 4. Case presentations

### 4.1. Case 1 with subclinical cardiac irAE

Case 1 was a 59-year-old man with metastatic gastric cancer who was previously treated with multiple regimens, including SOX (oxaliplatin in combination with S-1), paclitaxel, and apatinib. He had a history of hypertension for more than 4 years and was administered valsartan. In 2015 and 2018, he underwent coronary artery stenting without cardiac symptoms or laboratory abnormalities prior to immunotherapy. His baseline electrocardiogram, echocardiography, and cardiac markers were normal. Intravenous sintilimab (200 mg) was first administered with apatinib when his disease progressed again. Four weeks later, he complained of fatigue with rashes, and pruritus on his limbs and had an elevated TnI level (0.227 ng/ml). Electrocardiogram showed T wave changes, and echocardiography revealed pericardial effusion. He was diagnosed as immune checkpoint inhibitor-associated myocarditis and pericarditis. Immunotherapy was discontinued, and he was treated with methylprednisolone (initiation dose: 1 mg/kg/day). Laboratory tests showed that his TnI level had dropped to the normal range 10 days later. Subsequently, he continued to receive sintilimab infusions. Unfortunately, he died 4 months later because of disease progression and cachexia.

### 4.2. Case 2 with subclinical cardiac irAE

Case 2 was a 46-year-old female patient with breast carcinoma. Before treatment, any contraindications to ICIs were ruled out. She was enrolled in a phase 1 clinical trial and received PD-1 antibody infusions after surgery, radiation, and multiline chemotherapy regimens. Laboratory tests showed elevated levels of TnI (0.84 ng/ml) and LDH (393 U/L, reference range: 135–225) without any symptoms after 2 cycles of PD-1 antibody. Echocardiogram revealed trace pericardial effusion. On ECG, there were no unusual findings. She was diagnosed with pericarditis and myocarditis. She dropped out of the clinical trial and was treated with methylprednisolone (initiation dose: 2 mg/kg/day). One week later, her TnI and LDH levels had declined insignificantly. Her treatment was IVIG on a methylprednisolone treatment basis (Figures 1B,C). Ultimately, her TnI and LDH levels had dropped to the normal range 8 weeks later. The dose of methylprednisolone was reduced on schedule. Her PD-1 inhibitor infusion were not restarted, and she is not currently experiencing any discomfort.

### 4.3. Case 3 with acute heart failure

The third patient was an 85-year-old man with bladder urothelial carcinoma. He had a history of pulmonary heart disease for more than 10 years and received tislelizumab as the first-line treatment. Viral serology from peripheral blood was negative for Epstein‒Barr virus (EBV) and cytomegalovirus (CMV). He was negative for hepatitis B/hepatitis C and human immunodeficiency virus (HIV) before immune therapy. Three days after his first dose of tislelizumab, he presented with lower extremity edema and dyspnea on exertion. On our initial examination, his blood pressure was 61/44 mmHg, heart rate 101 beats per minute (bpm), temperature 36.2°C, respiratory rate 27/min and saturation 93% on 5 liters of oxygen *via* a nasal cannula. His total leucocyte count was 11.81 × 10^3^ cells/ml (reference range: 3.5–9.5 × 10^3^), hemoglobin 9.6 g/dl (reference range: 13–17.5), blood urea nitrogen (BUN) 18 mmol/L (reference range: 2.86–8.20), creatinine 206 µmol/L (reference range: 59–104), serum creatine kinase (CK) 602 U/L (reference range: 55–170), TnI 7.62 ng/ml (reference range: 0.0–1.0) and BNP 2,750 pg/ml (reference range: 0–100). His serum triglyceride and cholesterol levels were within the normal range. Electrocardiogram (ECG) showed sinus tachycardia with ST-T segment changes, an anterior myocardial infarction, and a conduction block in the left forearm (LAFB). Transthoracic echocardiogram at bedside was significant for a severely reduced LVEF of 18% from a baseline of 65%, a dilated left atrium cavity, a decreased diffusion of wall motion, mild pericardial effusion, and moderate tricuspid regurgitation.

The patient was started on milrinone *via* a pump and deslanoside injections. He was also placed on a norepinephrine drip to maintain a mean arterial pressure of 65 mmHg or more. Due to the left ventricular dysfunction in the setting of the recent ICI therapy, the possible diagnosis of cardiotoxicity was considered over acute coronary syndrome, and pulsed methylprednisolone (1,000 mg/day) was used for the treatment of the patient's cardiotoxicity. However, the patient did not show any signs of improvement while on inotropic support and corticosteroids and died 17 h after the onset.

### 4.4. Case 4 with arrhythmias

A 57-year-old male patient with metastatic lung squamous carcinoma who received gemcitabine/carboplatin and sintilimab on July 6, 2021 as the second-line setting after progressing through albumin-bound paclitaxel/cisplatin in another hospital, presented to our emergency department on July 24, 2021. Ten days after his first dose of sintilimab, he began to develop chest tightness and fever with a temperature of 37.5 and 38.5°C. His blood pressure was 80–90/45–60 mmHg, and he had a temperature of 37.8°C, a respiratory rate of 18 bpm, and an oxygen saturation of 92%. ECG showed a ventricular rate of 92 beats/min and ST-T segment changes. His total leucocyte count was 10.89 × 10^3^ cells/ml, hemoglobin 9.8 g/dl, serum CK-MB > 80 U/L, Tn I 23.7 ng/ml and BNP 60.8 pg/ml. Echocardiogram by bed presented decreased wall motion and pericardial effusion. He was clinically diagnosed as myocarditis and pericarditis. He was admitted to the cardiology department on July 25, 2021.

On admission, the patient complained of heart palpitations, accompanied by cold and wet sweating. ECG showed a ventricular tachycardia with a ventricular rate of 155 bpm, and his blood pressure could not be measured. The patient's oncologists and cardiologists worked together to develop a treatment plan. The patient was given assisted respiration immediately and was treated with vasopressor drugs (e.g., dopamine, norepinephrine), antiarrhythmic drugs (e.g., lidocaine and amiodarone), methylprednisolone (1 g/day) and intravenous gamma globulin at 400 mg/kg/day at the same time. However, the patient did not show any signs of improvement. Finally, he received electrical defibrillation therapy. Unfortunately, the patient died in the early hours of the morning of July 26, 2021 (12 days after his injection of sintilimab).

### 4.5. Case 5 with arrhythmias

A 71-year-old man with a history of diabetes mellitus was diagnosed with stage IIIA gastric adenocarcinoma. He received a radical operation and postoperative adjuvant chemotherapy based on fluorouracil regimens. Two years after surgery, a positron emission tomography-CT (PET-CT) revealed enlarged hilar lymph nodes with higher levels of glucose metabolism. Then, he received paclitaxel liposomes combined with a capecitabine regimen and tislelizumab. On Day 21 after tislelizumab initiation, he presented with systemic muscle pain accompanied by weakness and was admitted to our department. Labs showed CK-MB 250.4 U/L, troponin-I 8.3 ng/ml, Myohemoglobin (MYO) 2000 ng/ml (reference range: 0–121), lactic dehydrogenase (LDH) 1,993 U/L (reference range: 135–225), ALT 485.8 U/L, AST 1,093.8 U/l, and BNP 31 pg/ml. ECG showed a left anterior branch block and a complete right bundle branch block. An echocardiogram showed normal left ventricular wall motion with an LVEF of 60%. On Day 22 after tislelizumab initiation, ECG showed a III-degree atrioventricular block and premature ventricular beats. Chest radiograph showed bilateral pulmonary effusion and pleural effusion.

He was started on methylprednisolone 1,000 mg daily and IVIG for 3 days. Meanwhile, the patient underwent immediate implantation of a temporary pacemaker that was performed by a cardiologist. Symptom improvement was observed, the patient's laboratory indicators also declined, and the methylprednisolone dose was reduced to 240 mg daily on schedule. Bedside chest radiographs showed that the patient's bilateral lung exudation had improved, and his bilateral pleural effusion had decreased. However, seven days later, his heart rate had dropped (30–40 beats/min) and did not significantly improve after the administration of isoproterenol. The pacing voltage of the patient's temporary pacemaker was adjusted to 8.0 V. However, there was still leakage, and the patient's heart rate was 40–50 beats/min. Therefore, a temporary pacemaker implantation was performed again with the pacing rate adjusted to 60 beats/min after the implantation. He was discharged on prednisone after a period of intravenous steroid treatment. ECG showed no spontaneous rhythm throughout the entire period. Eventually, he had a permanent pacemaker implanted.

On a follow-up visit six months after the initial symptom onset, the patient denied having any episodes of muscle pain or weakness.

## 5. Discussion

Immunotherapy with ICIs improves the overall survival of patients with advanced or metastatic cancers and leads to irAEs such as cardiac toxicities. ICI-related cardiotoxicity is uncommon but critical with a high mortality rate and poor prognosis, especially for a small group of patients with symptomatic cardiac abnormalities. In other patients, there are subtle features of cardiac irAEs on the initial clinical presentations. Based on the results from this real-world study, more attention should be given to cardiotoxicity associated with ICIs, and these patients should have baseline examinations and biochemical analyses before and after the initiation of immunotherapy, intensive cardiac assessments, an accurate and rapid diagnosis, and timely multidisciplinary management with immunosuppressive agents and other clinical interventions to improve clinical outcomes.

The cardiac toxicities associated with ICIs include myocarditis, conducting system diseases, pericardial diseases, and vasculitis. Recent studies have demonstrated an incidence of myocarditis ranging from 0.06% to 1.1% (8, 9). Among those receiving combination therapy of CTLA-4 and PD-1 inhibitors, the risk of developing myocarditis is higher than single-agent therapy, and the severity of myocarditis appears to be increased (10–14). A systematic review showed that approximately 10% of the cardiotoxicity events associated with ICI therapy were atrioventricular block or conduction system disease, which leads to death in 50% of these patients (15). Pericardial diseases, vasculitis, hypertension, symptomatic sinus tachycardia, angina pectoris, and Takotusbo-like cardiomyopathy have been sporadically reported (16–18). However, our case series only included patients with myocarditis. In our study, the incidence of cardiac irAEs was as high as 2.46%, which was higher than the previously reported percentage ranging from 0.06% to 1.1% and this could be due to possible or unconfirmed myocarditis. Nevertheless, cardiac biopsy and CMR are difficult to perform because of the technical difficulty or patients' poor clinical status.

The clinical symptoms of immune-related cardiovascular toxicity are varied and may manifest as mild nonspecific symptoms such as fatigue and weakness. Most commonly, patients with ICI-related cardiotoxicity have a primary complaint of shortness of breath (19). In severe cases, patients present with dyspnea, chest pain, acute heart failure, pulmonary edema, bilateral lower limb edema, atrial fibrillation, other supraventricular arrhythmias, ventricular tachycardia, ventricular fibrillation, and conduction delays, including complete heart block (20, 21). Atrioventricular block and atrial fibrillation may be secondary to inflammatory infiltration of the myocardium (22, 23). ICI-related cardiotoxicity can be diagnosed in the following ways: (1) the combination of an elevated TnI level and the presence of late gadolinium enhancement on a cardiac magnetic resonance study in a pattern typical for myocarditis and without evidence of coronary ischemia on standard testing and (2) the presence of typical cardiovascular symptoms, congestive heart failure, an elevated troponin, a reduced LVEF, and without evidence of coronary ischemia using coronary angiography in patients who did not have a biopsy or cardiac MRI (21). Invasive heart biopsy is extremely difficult to conduct. In addition, cardiac MRI also has many risks and limitations. In these 12 patients, the diagnosis was based mainly based on the latter because of the risks and limitations of cardiac biopsy and CMR. No associations have been found between myocarditis and a speciﬁc type of cancer. Up to 25% of patients with myocarditis may have concomitant myositis, and 10% may have concomitant myasthenia gravis (24). In our study, 4 patients with myocarditis (22%) developed concurrent myositis, and 3 patients with severe cardiac irAEs presented with myasthenia gravis-like symptoms such as blepharoptosis. Two patients had symptoms of weakness in the limb girdle and axial distribution.

The exact mechanism of cardiac irAEs is not entirely clear, but cross-reactive T cells (T cells that bind to both tumor and cardiac tissues) may play a role. An autopsy study in two patients with melanoma who both had died from fatal myocarditis and myositis after treatment with a combination of CTLA-4 and PD-1 inhibitors showed shared T-cell clones in the tumor, heart, and skeletal muscle but not in smooth muscle (21). A study confirmed that nivolumab increases proinﬂammatory cytokine production, including TNF-α, granzyme B, and IFN-γ, in CD4+ T cells but does not induce cardiomyocyte apoptosis (25, 26). In all patients with myocarditis, various stressors could have a contributing role (27, 28).

The risk factors for acute cardiovascular irAEs are unknown (29). A multicenter study showed that patients with ICI-associated myocarditis had multiorgan irAEs with a high incidence of severe myocarditis, mortality, and poor prognosis (30). In an observational cohort study of 35 patients with ICI-associated myocarditis, most had an elevated TnI level and abnormal results on electrocardiogram, but half had of them had a preserved LVEF. A TnT level ≥1.5 ng/ml was associated with a fourfold increase in major cardiac irAEs during follow-up (22). Concomitant myocarditis and myasthenia gravis-like symptoms are common, and the case fatality rates of concomitant myocarditis and myasthenia gravis-like symptoms are higher than with other irAEs (31). Patients with higher levels of CRP in the plasma have more atrial fibrillation episodes, and baseline plasma CRP levels are predictive of the future risk of atrial fibrillation (32). A real-world investigation involving 495 cancer patients with ICIs showed that PD-1 inhibitors had higher cardiovascular adverse event occurrences than PD-L1 inhibitors (33). In our report, CRP was elevated in 8 patients (66.7%), 6 of whom had systemic symptoms, and all patients with myocarditis received a PD-1 inhibitor but not PD-L1 inhibitor treatment. Moreover, access to detailed clinical and laboratory information, as well as uniform follow-up monitoring for cardiotoxicity related to ICIs, cancer progression, and death, allowed us to estimate the cumulative incidence and risk factors for irAEs.

Due to nonspecific and overlapping manifestations and difficulty in etiological diagnosis, classification based on clinical presentation and severity is more appropriate for occupational therapists in most centers. The subclinical type of cardiotoxicity manifests as a variety of abnormal biomarkers (mainly TnI, CK, BNP) with no symptoms. ICI-induced asymptomatic myocarditis can occur with concurrent myositis or myasthenia gravis in some cases (5, 6, 34). In the present study, the incidence of cardiac irAEs was as high as 2.46% (subclinical type 0.82%; severe type 1.64%) in the single center. This may suggest that cardiotoxicity may be more common than appreciated. Of concern, 50% of the ICI-associated myocarditis cases were fatal. Severe cardiac toxicities can occur as soon as immediately after the first ICI dose, and the median time is 30 days (range: 18 to 60) after the initial exposure to ICIs, which was earlier than mild cases (11). The data from our center show that the mortality of ICI-related cardiotoxicity was 25.0% and 62.5% in severe types. The median time was 33.5 days (range: 20.3 to 46.8 days), with 83.3% of these ICI-related cardiotoxicities presenting within 3 months. The median onset time of severe cases was 24.5 days (range: 16.0 to 39.8 days), which was consistent with previous literature reports.

Although patients with subclinical-type cardiac irAEs have a favorable prognosis and mortality is significantly increased in severe types, we do not know whether patients can quickly progress from subclinical-type to clinical-type cardiac irAEs. Our data showed that the median time from the initial immunotherapy to the onset of cardiac irAEs for the subclinical type was longer than that for the clinical type. The initial evaluation should include a detailed history and physical exam to elicit signs and symptoms of active cardiac disease. Early identification of these subclinical types and early intervention should be performed to avoid serious adverse events. That is, we must focus on baseline examinations and biochemical analyses before the initiation of ICIs, obtain an accurate and rapid diagnosis, and perform timely multidisciplinary management with immunosuppressive agents and other clinical interventions. In the follow-up period, none of the patients developed recurrent cardiotoxicity after the initial cardiotoxicity event.

Given current knowledge, the key to managing cardiac irAEs is early recognition, interrupting ICI administration, and intervention with systemic corticosteroids. It is involved in cardiology, oncology, and cardio-oncology. In addition to interrupting ICI administration (temporarily for subclinical cases or permanently for severe cases), the mainstay of treatment is corticosteroids, but intravenous immunoglobulin, mycophenolate, inﬂiximab, tacrolimus, and antithymocyte globulin can also be administered. A higher initial steroid dose was associated with a lower rate of major adverse cardiac events (21). For severe cases, pulse cortisol therapy (500–1,000 mg/day intravenous methylprednisolone) is indicated for 5–7 days until clinical stability is achieved, followed by oral prednisolone at 1 mg/kg/day initially according to the response. For subclinical types, oral prednisolone at 0.5–2 mg/kg/day initially can be recommended according to the symptoms. Meanwhile, conventional cardiac treatments cannot be ignored in all cases and include the treatment of acute heart failure and pulmonary edema with intravenous nitrates and diuretics, the implantation of cardiac pacemakers for complete heart block, treatment with *β* blockers or amiodarone for ventricular tachyarrhythmias with external cardioversion, or defibrillation for hemodynamically unstable ventricular tachycardia and ventricular fibrillation (10). However, in this study, death still occurred in 1 patient who received an initial dose of methylprednisolone of 1,000 mg. Tacrolimus was used in 1 patient with successful treatment. After the initiation of corticosteroids and guideline-conforming heart therapy, the symptoms for all patients rapidly improved. In all patients, the CK-MB levels rapidly normalized in one month, but the TnI levels usually took several months to normalize, which is in line with a recent report (35). ICI-mediated cardiotoxicity may be life-threatening, but the surveillance strategy is immature, with a risk of blindness. Even in subclinical cases diagnosed by surveillance, specialist cardio-oncology review is recommended.

We acknowledge the limitations of the study, including its retrospective nature and small sample sizes, and it is not a multicenter study and does not include prospective data. The incidence was relatively high and could not fully reflect the actual situation, as some included patients were admitted for ICI-related cardiotoxicity but were initially diagnosed in other centers. Additionally, there are still defects in the diagnosis of ICI-related cardiotoxicities, including asymptomatic myocarditis, due to the lack of a uniform standard. In this study, the diagnosis of ICI-related cardiotoxicity was viewed as a clinical diagnosis that depended mainly on clinical features, including the signs and symptoms, biomarkers, electrocardiogram and echocardiography findings, prior medication history of ICIs, and response to steroid treatment. In particular, a prior medication history of ICIs is very important, and ICIs are associated with the occurrence of immune-related myocarditis, which has a high mortality of nearly 50%. TnI is the most sensitive and specific biochemical marker of asymptomatic myocardial injury. TnI elevations are also found in many disease states, including cancer, and do not necessarily indicate the presence of concurrent coronary disease or ICI-related cardiotoxicity (36). Peri-myocarditis due to coronary disease, cancer invasion or chronic kidney disease was excluded in these patients *via* clinical evaluation. Other anticancer treatments and infections could be associated with myocarditis. Recently, several suspected cases of COVID-19 myocarditis have been reported (37), and even the incidence of cardiovascular complications in cancer patients with COVID-19 is expected to be high (38). However, no similar cancer patients with nonirAE myocarditis were found in our cohort. Future studies should include more cancer or noncancer patients with nonirAE myocarditis. Last, there are differences in the level of multidisciplinary management of cardiotoxicity, even with the guidelines. More high-quality trials with large samples and longer follow-up are proposed in the future.

## 6. Conclusion

Cardiotoxicity related to ICIs is relatively rare but can be serious and potentially fatal. Clinical comprehensive management requires careful coordination between oncology and cardiology specialists. More attention should be given to cardiotoxicity associated with ICIs, and these patients should undergo baseline examinations and biochemical analyses before and after the initiation of immunotherapy, intensive cardiac assessments, and timely multidisciplinary management with immunosuppressive agents and other clinical interventions to improve clinical outcomes.

## Data Availability

The original contributions presented in the study are included in the article/Supplementary material, further inquiries can be directed to the corresponding author.
